# Medical Relief in London

**Published:** 1890-04-19

**Authors:** 


### Passing Topics.

### MEDICAL RELIEF IN LONDON.

Under this heading Mr. C. S. Loch contributes to the current number of "Murray's Magazine," a paper called forth apparently by the recent intimation that the Government are willing to consent to the appointment of a Select Committee to inquire into "the financial and general management and common organization of medical institutions in the metropolis."

While cordially assenting to the desirability of an inquiry, and believing that the deserving institutions have nothing to fear from it, we have not ceased to regret that the demand for the inquiry did not emanate from the hospitals, and that its promotion should have been left to the Charity Organization Society, a body which, if it has so far favoured the hospitals as to make a somewhat free use of them, has none the less shown itself consistently out of sympathy with their wants, their work, and their aims.

The petition for the inquiry was nothing but a bill of indictment, and if the promoters indulge in some self-congratulation at the success of their manœuvres, it is gratifying to note in the article before us a disposition—we will not doubt its sincerity—to deal more fairly with the institutions for which the wit of man has been as yet unable to devise a substitute, and to acknowledge that the "defects" set forth in the petition may occupy "a lesser place and proper proportion," beside the records which show the present to be as it is, "the nearer rim and fringe of a splendid past."

It is true that when we read on we find this tribute is rendered to the hospitals as vehicles of medical and surgical education, rather than in their capacity of philanthropic institutions, whose chief business is to assuage suffering; but we must not express wonder that a body with the history and traditions of the Charity Organization Society should inspire its representative with a distrust of mere benevolence and render him incapable of realizing a fact patent to every hospital worker—that the men and women who support our medical institutions yield to their compassion what they would deny to the most prodigal promises of science.

It is the inability to estimate the real motives actuating human nature as distinguished from the artificial contrivances retailed in the Adelphi, which causes so much of the energy of the Charity Organization Society to run to waste. People of truly benevolent instincts will not wait to adjust a piece of complicated mechanism warranted to test the merits of a case which appeals to their sympathies when its necessities are too obvious to be overlooked.

Mr. Loch entirely fails to make any fresh points against the hospitals, and has to content himself with urging the old complaints—that they are pauperizers, that they lack uniformity of method and expenditure, that their work is inimical to the general practitioner, and that there are too many special hospitals. We are as ready as the writer to urge some of these complaints, and to admit that the out-patients of our general hospitals are far too numerous, but we refuse to believe that because this is so, it is already as good as settled that the provision made by our hospitals is in excess of the requirements. On the contrary, however shocking the proposition may be to the "organized," we assert that the provision is in many ways lamentably insufficient, and that certain classes of sufferers are virtually left to their doom.

Mr. Loch puts the number of beds in the voluntary hospitals at 8,888, with an average of 6,371 in occupation. We think these figures are too liberal, but, accepting them as correct, can it be said that they represent an excessive provision for accidents, and the graver maladies among the poor of a population approaching five millions, plus the many country patients attracted to the metropolis? Space does not permit us to pursue the examination of Mr. Loch's paper. We agree with him in welcoming the inquiry, but it must be an independent one. That the hospitals should have been adroitly placed in the position of defendants is unfortunate, but at least it must be insisted that the Charity Organization Society shall not be both judge and complainant. We cannot afford to allow these splendid institutions, built up by the efforts and sacrifices of a noble philanthropy, to be despoiled or discredited at the bidding of an irresponsible society with a craze for meddling, and the Government will do well to dissociate the inquiry from a suspicion that it is intended to register a foregone conclusion. Agricola.

